# Why Collaborative Care for Depressed Patients is so Difficult: A Belgian Qualitative Study

**DOI:** 10.5334/ijic.2491

**Published:** 2017-06-21

**Authors:** Kris Van den Broeck, Frédéric Ketterer, Roy Remmen, Marc Vanmeerbeek, Marianne Destoop, Geert Dom

**Affiliations:** 1 Collaborative Antwerp Psychiatric Research Institute, University of Antwerp, Antwerp, BE; 2 Département de Médecine Générale, University of Liège, Liège, BE; 3 General Practice, Department of Primary and Interdisciplinary Care, University of Antwerp, Antwerp, BE; 4 Psychiatric Centre Brothers Alexianen, Boechout, BE

**Keywords:** collaborative care, major depressive disorder, primary care, general practice, mental health services, qualitative study

## Abstract

Although current guidelines recommend collaborative care for severely depressed patients, few patients get adequate treatment. In this study we aimed to identify the thresholds for interdisciplinary collaboration amongst practitioners when treating severely depressed patients. In addition, we aimed to identify specific and feasible steps that may add to improved collaboration amongst first and second level Belgian health care providers when treating depressed patients. In two standard focus groups (n = 8; n = 12), general practitioners and psychiatrists first outlined current practice and its shortcomings. In a next phase, the same participants were gathered in nominal groups to identify and prioritise steps that could give rise to improved collaboration. Thematic analyses were performed. Though some barriers for interdisciplinary collaboration may seem easy to overcome, participants stressed the importance of certain boundary conditions on a macro- (e.g., financing of care, secure communication technology) and meso-level (e.g., support for first level practitioner). Findings are discussed against the background of frameworks on collaboration in healthcare and recent developments in mental health care.

## Introduction

Major depressive disorder (MDD, [1]) is highly prevalent [2] and its personal and societal impact is significant [3]. Though effective treatment interventions exist, it is estimated that only half of the depressed patients in Europe receive adequate treatment within an acceptable timeframe [4]. Different factors contribute to this treatment gap, i.e., a substantial portion of depressed patients is reluctant to seek help [5,6], and some depressive episodes are misdiagnosed or remain unrecognized [7]. But even those patients who are correctly diagnosed, often do not get the most effective treatment. For instance, current evidence-based guidelines [8,9,10,11,12] agree that severely depressed patients should be treated with both pharmacotherapy and psychotherapy. In addition, treatment should be multi-professional, involving the patient’s general practitioner (GP) and at least one other health professional, and health care providers should enhance their interprofessional communication. Yet, a recent study reveals that only 51% of the severely depressed (Belgian) patients is referred to a mental health professional, and that only 34% of them receives both antidepressants and psychotherapy [13]. Thus, the ‘collaborative care’ paradigm (CC, [14]) – as this multi-professional approach is generally referred to – seems to be insufficiently realised within the current practices.

These observations raise the question which elements add to a successful implementation of a CC programme, and additionally, once a programme is installed, what factors should be taken care of in order for practitioners to sustain such a programme. Based on a systematic review of the current guidelines regarding MDD, Van den Broeck, Remmen, Vanmeerbeek, Destoop and Dom [15] conclude that guidelines nowadays provide few concrete directives on the organisation and maintenance of CC. Authors that have looked at factors impeding or facilitating the implementation (or maintainance) of a CC programme (for depression, but also for other illnesses) generally agree that cultural and structural adaptations are necessary in order to successfully install and sustain such a programme. For instance, the attitude of both policy makers [16,17,18] and professionals [19,20] is thought to be critical when switching to a model in which health care professionals more closely collaborate with each other. Furthermore, according to Hall [21], true collaboration requires professionals to be able to discuss with each other on an equal basis, but professionals today are not considered equal regarding responsibilities and status.

A number of structural facets may be helpful as well to initiate or sustain a CC programme. Some authors stress the need of education, e.g. in order to develop a common vocabulary across professions [21], in order to train professionals in the intervention [22], or in order to inform and involve specialists who are not familiar with the illness or CC [23]. Others emphasize that the availability of financial resources for supporting the initiation and continuation of a CC programme may increase the chances of success [18, 22]. Another driver concerns well-developed arrangements about insurance and/or reimbursement for patients [23, 24]. Finally, on a more daily basis, the installation of a CC programme is thought to benefit from practical support to professionals, e.g., to help them integrating each other’s workflows, to change their roles and job descriptions, to facilitate the way they communicate with each other, etc. [18, 24]. Specifically in relation to mental disorders, Lester [25] states that both GPs and psychiatrists may benefit from clarity about the roles and tasks they are expected to be involved in. Furthermore, collaboration would improve from ameliorated communication from both sides.

Previous studies clearly suggest that the success of a CC programme largely depends on local policy and culture. The first aim of this study therefore is to come to a clear description about how Belgian professionals collaborate around severely depressed patients today. Furthermore, although many co-determinants of CC have already been identified, we think practice could benefit from a more in-depth analysis exploring the inducements of good or failed interdisciplinary collaboration in everyday practice. For instance, what is missing to effectively collaborate? Or, are there any issues that should be solved in order to ameliorate collaboration? Thus, in sum, the aim of our research is twofold: We aim (1) to describe how (Belgian) practitioners currently work together when treating depressed patients and what barriers and good practices they experienced so far; and (2) to identify which steps they believe could be taken to improve collaboration amongst professionals.

## Methods

This study was performed in Belgium. This small country has a Dutch speaking and a French speaking region. Organisation of health care is largely identical in both regions, but because of the differences in language, we choose to organise separate group discussions. Combining convenience and snowball sampling,^1^ we sought to create a balanced group of GPs and psychiatrists in each region. We selected participants that worked in or nearby a large city (Antwerp or Liège, for Dutch- and French speaking areas, respectively; see Table 1 for respondents’ characteristics). Participants were invited by phone and e-mail, and gave oral and written informed consent before participating in the study. For each group discussion they participated in, they were rewarded with vouchers worth €30.

**Table 1:** Participants’ characteristics.

Code	Sex	Provides ambulatory care	Provides residential care	Participated at nominal group

dGP1	Female	Group practice	NA	Yes
dGP2	Female	Group practice	NA	Yes
dGP3	Male	Solo practice	NA	Yes
dGP4	Male	Solo practice	NA	No
dPSY1	Male	Private practice + ambulatory mental health service	No	Yes
dPSY2	Male	Private practice + outpatients while based at a psychiatric hospital	Limitedly	Yes
dPSY3	Male	No	Psychiatric hospital	Yes
dPSY4	Male	No	Psychiatric ward of a general hospital	No
fGP1(FB)	Female	Group practice	NA	Yes
fGP2(AS)	Female	Community health centre	NA	Yes
fGP3(AM)	Female	Community health centre	NA	No
fGP4(MS)	Female	Solo practice	NA	No
fGP5(NW)	Female	Solo practice	NA	Yes
fGP6(AD)	Male	Solo practice	NA	Yes
fGP7(JF)	Male	Group practice	NA	Yes
fPSY1(DG)	Female	Outpatients while based at a psychiatric hospital	Limitedly in a psychiatric hospital + psychiatric ward of a general hospital	No
fPSY2(SS)	Female	Private practice	Psychiatric ward of a general hospital	Yes
fPSY3(JLK)	Male	Outreach	Psychiatric hospital	Yes
fPSY4(DB)	Male	Private practice + outpatients while based at a psychiatric hospital	No	Yes
fPSt1(NB)	Male	Day care	No	Yes

A participant’s code is consisted as follows: d = Dutch speaking; f = French speaking; GP = general practitioner; PSY = psychiatrist; PSt = Psychiatrist trainee.

A two stage method was used. Using multidisciplinary focus group discussions, we first investigated how GPs and psychiatrists currently experience interdisciplinary collaboration in everyday practice during all treatment phases. These discussions were organised in October 2015. To avoid misunderstanding and to be sure all participants discussed the same phenomenon, we clearly defined MDD at the beginning of the focus groups using a free video (with permission of the author, Stomp on Step 1 [26], 00:30–06:00, French and Dutch subtitles were used), which referred to the DSM-5 criteria for MDD (APA, 2013). Afterwards, participants were asked to report on good and bad experiences regarding interdisciplinary collaboration when dealing with severely depressed patients.

The discussions were recorded and transcribed. Data were analysed using Nvivo 11.0. We used open and axial coding and conducted a thematic analysis. Additionally, each fragment was assigned to a treatment phase (treatment in general – diagnosis and referral – treatment – follow-up), which allowed us to construct an overview of potential barriers and facilitators over the course of treatment.

Subsequently, in January 2016, we organised two nominal groups (one in each language part, see, e.g., [27]) with the same practitioners invited in the previous groups. In these structured follow-up discussions, participants were asked to generate and prioritise suggestions to improve collaboration. Following a summary of the findings of the previous group discussions, participants were given 20 minutes to individually write down answers to the following question: ‘Which feasible changes, meeting the expectations of both GPs and psychiatrists, may result in an improved collaboration amongst GPs and secondary mental health practitioners when treating severely depressed patients?’. Afterwards, all answers were inventoried, and some answers were joined together by consensus. At the end of the session, each participant chose the five propositions he or she thought were the most valuable ones. The personal favourite was awarded with five points, the next one with four, and so on. Propositions with the highest scores over all participants can be considered the ones with the highest priority, whereas propositions that received more votes are considered more popular than others.^2^ Also, each proposition was assigned to one of the previously defined treatment phases during analysis (treatment in general – diagnosis and referral – treatment – follow-up).

The studies were approved by the ethical committees of both Antwerp (ref. 15/38/401) and Liège Universities (ref. 2015/216).

## Results

We organised our findings according to the treatment phase to which they are applicable. Each of the following paragraphs starts with a summary of the information we retrieved from the standard focus group discussions about current interdisciplinary collaboration (including experienced barriers or facilitating factors), subsequently completed with the most important suggestions for improvement proposed in the nominal group discussions. Table 2 summarizes the proposals at the micro (interventions situated within the actual treatment trajectory), meso (interventions supporting collaboration amongst practitioners) and macro levels (suggestions pointing at conditions necessary in order to install CC).

**Table 2:** Summary of suggestions to improve collaboration amongst practitioners when dealing with severely depressed patients, classified by system level.


**Suggestions at macro level: conditions necessary to install collaborative care** Safe and easy-to-use technology to support communication Clarity about professional confidentiality Reimbursement of psychotherapy provided by psychologists Adapted nomenclature
**Suggestions at meso level: measures supporting collaborative care** Knowing each other (both formal and informal) Small-scale networks with steady partners Support for GPs in terms of education, a help line, an up-to-date (online?) tool, presenting an overview of all care taking facilities and health care providers
**Suggestions at micro level: possible to implement rapidly** Professionals should make arrangements regarding reachability and availability Professionals should make arrangements about how and when what to communicate Professionals should make arrangements about each other’s roles and tasks Professionals should set up intervision moments to discuss current practice and collaboration Professionals should include case management and monitoring in daily care for severely depressed patients


## Treatment in general

GPs said that they did not often see severely depressed patients, but they were regularly confronted with what they call ‘urgent cases’: (potentially) aggressive patients who cannot easily get motivated for referral. GPs then feel helpless. Psychiatrists responded that crises (periods of intense difficulty) are more probable than emergencies (situations that need immediate attention/action). When referral was an option, GPs often were confronted with waiting lists at the psychiatrist’s consultations. Knowing each other was thought to be beneficial for collaboration: It is easier to contact one another as one knows each other’s strengths. Participants found that the potential position and role of clinical psychologists in the treatment of severely depressed patients was unclear. All practitioners supported the idea that therapies offered by psychologists should get financially reimbursed (which was not the case at the time these studies were conducted). Finally, practitioners believed that the presence of a safe, electronic communication system would facilitate communication amongst health care providers and improve continuity of care.

dPSY4: “We talked a lot about communication, and I strongly believe that a fluent, electronic way of communicating with each other would be of great help. […] So, if I discharge someone from the hospital today, his GP instantly knows: ‘this patient is at home, his current medication is that and that, the following agreements were made’.”

Some suggestions formulated during the nominal groups closely related to the themes that came across during the standard focus groups. Technology related propositions were formulated in both nominal groups, and got votes from both GPs and psychiatrists. A safe and easy-to-use shared electronic patient file had the highest priority of all propositions. Furthermore, six propositions were related to the position of clinical psychologists. Reimbursement of psychotherapy by clinical psychologists received high priority with 5.6% of all points, all awarded by GPs. Other important propositions concerned the encouragement of personal contacts (of all kind) amongst health care providers, and the suggestion to treat MDD as a chronic illness (e.g., included case management). ‘Counter referral’ for psychiatric patients, by which treatment is initiated by the psychiatrist and stabilised patients are referred to the GP, was an original idea. It was thought to discharge GPs from ‘difficult’ patients, but it is unclear whether efficiency of care will improve.

## Diagnosis and referral

Largely in line with what is included in the guidelines, GPs stated in the focus groups that they referred patients to a psychiatrist in case of acute suicidality, psychotic elements, increased distress, and following one or more failed attempts to relieve the patient’s complaints. According to psychiatrists, GPs in this phase should (1) diagnose MDD if present, and assess its severity; (2) motivate patients for referral if indicated; and (3) properly prepare the referral. A detailed anamnesis and a concrete question or expectations addressed to the psychiatrist was believed to shorten the psychiatric intervention. GPs stated that easy accessibility of a second level mental health professional may facilitate referral, whereas waiting lists, limited knowledge about referral possibilities, and the patient’s reluctance for referral sometimes impeded it. GPs then tended to initiate pharmacotherapy, but psychiatrists argued that GPs’ knowledge about antidepressants was limited, resulting in misuse of antidepressants. First level psychologists (who operate nearby a GP’s practice and offer short trajectories of counselling or therapy) and Flexible Assertive Community Treatment (FACT-) teams were generally experienced as very supportive by GPs, who often declared a lack of knowledge about psychotherapy and found it difficult to judge the quality of psychotherapists.

dGP4: “I sometimes receive a letter from a newly started psychologist, stating which psychotherapy courses he followed, but at the end, that does not tell me much.”

dGP3: “There is a lot of variety of skills and competencies within the group of psychologists and psychiatrists.”

During the nominal groups, the most important propositions concerning this treatment stage were related to fast transfer of patients (psychiatrists should reserve slots for patients referred by the GP; there should be a direct line for patients who are in need of residential care, and care should be taken over immediately), supportive measures for the GP (an up-to-date tool, presenting an overview of all care taking facilities and health care providers; a central help function for GPs), and an improved and systematic way of referring patients (asking a concrete question towards the psychiatrist when referring a patient; handing over a detailed anamnesis and information about the patient’s current medication).

## Treatment

GPs regretted that professional communication with psychiatrists in this phase of treatment was often limited. They would like to be briefly informed at the start of therapy by the psychiatrist (and not only by the patient) about their patients’ diagnosis, the aims of treatment, and important health care providers (and their contacts) involved. During treatment, GPs would like to be kept (briefly) informed on a regular basis about their patients’ compliance and evolution. Furthermore, GPs insisted on interdisciplinary contact when the psychiatrist was not able to meet the GP’s demand or expectations, or in case of unexpected twists during treatment. GPs, from their position, may also provide interesting information about the patient, since they generally know the patient already longer. According to psychiatrists, patients sometimes forbid psychiatrists to share information with their GP, every now and then because patients doubt the professional confidentiality of a GP. Nevertheless, GPs thought it was important to be informed about the medication their patients used. Finally, practitioners agreed that health care providers’ roles and tasks were not always clearly defined. Agreements should be made, for instance, about who is responsible for medication prescription.

When more than one professional is involved.

fPSt1: “[…] I often contact the GP to complete a medical history when there are items that are a bit weird or that I do not understand. The input of the GP is extremely valuable, even in psychiatry, and especially when the GP really knows the history of the patient and his family. […] Sometimes, GPs call me to ask about details of a hospitalisation they do not understand. I think this encourages collaboration, and it is true that the phone is very efficient, it is faster, you do not have to write an entire letter.”

dPSY2: “Not every patient has full confidence in the professional confidentiality of his GP, who also sees his father and his mother and so on. This is what I sometimes pick up, and I definitely take this into account in my work.”

fGP6: “We need at least some objective information, because a patient can always tell us what he wants, e.g., when he saw the psychiatrist, if in fact his appointment was postponed or whether he could not attend, etc., and what was actually prescribed as a chronic treatment. Now, that’s a real problem, I think. And it is not always obvious. Assume it is Friday evening, 19h, and the pharmacy is closed. When did he got his last prescription for benzos? Is there a risk when I re-prescribe?”

Priority scores of the propositions related to this phase of treatment were rather low. These propositions again closely related to what has been suggested already during the standard focus group discussions. Only one proposition was supported by both professions: Psychiatrists should more systematically communicate with GPs, especially at the start and at the end of treatment. In case a patient is hospitalised, GPs preferred to be informed before discharge, giving them the opportunity to organise home treatment if necessary.

## Back-referral and follow-up

According to most psychiatrists, patients in remission should be referred back to the GP, though some French speaking psychiatrists stressed the importance of regular psychiatric follow-up for some patients. Back-referral was thought to be complicated if a patient has become attached to the psychiatrist (or vice versa). This in turn may result in extended waiting lists. GPs expected to get informed at the end of treatment (or shortly before) by the psychiatrist, at least about the medication their patient uses (dosage, planned continuation of medication), any follow-up appointments the patient has with his psychiatrist and any special concerns the patient needs. Meetings in which professionals (together with the patient) discuss the coordination and continuation of care were considered an interesting way to successfully install follow-up. However, such meetings are seldomly organised, because they are time consuming. In general, psychiatrists expected GPs to become the patient’s case manager in this phase. He should organise and coordinate the care around the formerly depressed patient.

Few propositions to improve current practice during this phase of treatment were made, and their scores were rather low. Both French and Dutch speaking practitioners stressed the importance of communication amongst health care providers to improve treatment during follow-up. This could be done by the former mentioned coordination meetings, by developing a patient-specific crisis plan, or by agreeing on the tasks each practitioner should take care of.

## Discussion

### Main findings

The current studies aimed (1) to describe how Belgian practitioners currently work together when treating severely depressed patients and to deepen out the barriers and good practices they experienced so far; and (2) to identify what could be done to increase collaboration amongst health care providers, so practice will become more in line with what is recommended by current guidelines on MDD. Taken together, our findings suggest that collaborative care regarding severely depressed patients today is limited in Belgium. According to our participants, collaboration is impeded, at least in part, because important resources (e.g., technological support, financial arrangements for psychologists) are lacking, and because there are unresolved deontological issues, indistinct roles, and divergent conceptualizations of depression.

More specifically, the focus groups revealed that role definition (e.g., who should be the case manager?) and task ownership (e.g., who prescribes the medication: the GP or the psychiatrist? What responsibilities may be taken by the newly recognized psychologist?) were often unclear or were not fully respected. In particular, we noticed that practitioners tended to define each other’s roles – GPs missed psychiatric support during referral (e.g., no slots) or treatment (e.g., regular updates) and afterwards (e.g., follow-up of medication), whereas psychiatrists stated that GPs may add to collaborative care by referring their patients with all relevant information (e.g., anamnesis, aim of referral), and by coordinating care during follow-up – rather than reflecting on potential behavioural changes they could do theirselves to improve collaboration. Regarding the nominal groups, practitioners’ most important suggestions for improvement (top 5) were: (1) A common, safe and easy-to use electronic patient file; (2) considering severe mental illnesses as chronic illnesses and treating them alike (care pathway); (3) reimbursing psychotherapy delivered by (acknowledged) psychologists; (4) using the existing regional e-health networks more often; (5) improving interpersonal relationships amongst practitioners (all kinds of initiatives). It is remarkable that these were not situated within the actual treatment trajectory (micro level; referring to changes that may be relatively easy to implement in daily practice; see also Table 2). Rather, they referred to necessary conditions for (macro level) or interventions supporting optimal collaboration amongst health care providers at the meso level. Additionally, overall, our studies pointed out that lack of clarity regarding the sharing of information, which also involves the patient’s right to keep information private, and lack of a user-friendly, safe and practical way to communicate with each other, are thought to complicate collaboration, whereas knowing each other well was thought to lower the barrier to work together. Finally, we have the impression that GPs and psychiatrists often talk cross each other when they discuss psychiatric conditions (e.g., in contrast to psychiatrists, GPs often experience a sense of urgency when dealing with psychiatric conditions, and given the prevalence of MDD, we think GPs may underestimate the number of (severely) depressed patients in their practice). This, of course, also complicates collaboration amongst practitioners.

### Further reflections towards improvements in the field

The practitioners identified reasons why collaborative care is so difficult. Yet, as practitioners seemingly expect the others to change their behaviour to improve collaboration, we could additionally question the willingness of practitioners to collaborate. Indeed, the statements of psychiatrists doubting the professional confidentiality of GPs potentially reflect a credibility gap. GPs on the other hand, clearly ask the psychiatrists to share the care regarding psychiatric patients.

In line with our findings, studies investigating collaboration and its determinants in other areas of healthcare [e.g., 16,17] also showed that practitioners’ ability and willingness to collaborate strongly depend on external support (cf. the elements we identified at macro and meso level), as well as reciprocal trust (secured in agreements, cf. the elements at micro level). According to de Rijk et al. [17], actors’ divergent desires to collaborate may reflect differences in their goals (e.g., the psychiatrist’s goal may be to tackle the depressive episode, whereas the GP’s goal on the longer run is to take care of his patient’s health), as well as the extent to which an actor perceives oneself as dependent from the other actor to reach one’s goal. This would explain why GPs, who generally have consistent relationships with their patients over time and report to feel less confident when treating psychiatric patients, may be more willing to collaborate than psychiatrists, who are able to treat patients independently from the GP (which is sometimes even requested by the patient) and whose interventions may be relatively limited in time. Alternatively, as already mentioned above, these observations may reflect the inequality between first and second level practitioners [e.g., 21].

Taken from another angle, our present and previous findings [e.g., 15, 17] suggest that the provision of resources and a changed relationship (in terms of the perception of dependency and common goals) between practitioners would give rise to more collaborative care. However, regarding practitioners’ (greatest) desire to have a safe electronic communication channel, we believe that its presence alone would not automatically result in an improved collaboration amongst practitioners. Though both practitioners and patients consider electronic information exchange in healthcare to result in better quality and more efficiency of care, important prerequisites to be used widely concern the accessibility and usability of such an application, and its accurateness in terms of technology and privacy [e.g., 28,29]. Moreover, it should not disrupt the workflow of practitioners and it should contain additional information [28, 30]. The latter, we think, requires practitioners – again – to change their attitude of mind. As already outlined in the introduction [e.g., 21, 23], medical schools may have an important responsibility to integrate interdisciplinary training and models of collaborative care in the current education of our (future) doctors. Furthermore, frameworks on collaboration emphasize the necessity of governance and management when installing collaboration amongst health care providers [16,17,18]. Besides offering resources, managers and policy makers (and again, medical schools) should facilitate the process towards improved collaboration by aligning the practitioners’ perceptions and goals, and by sharpening their skills to work together. In sum, these frameworks suggest that improvements regarding collaborative care should be made together with the actors of the field, but not solely by them.

In this regard, it is also worth mentioning that the profession of clinical psychologists is recently properly regulated in Belgium, and proposals have been worked out to reorganise the reimbursement system allowing better access for psychotherapy [31]. In addition, the Flemish minister of health and social affairs recently introduced pilot studies in which psychologists are funded to work integrated within GPs’ practices. This allows psychologists to gain a spot in collaborating networks of physicians and stimulates the discussion about roles and tasks of all collaborating partners.

### Limitations and strengths

Qualitative research should be interpreted with care, given that the background of the interviewers may strongly determine the output of the study. Although the main analyses for these studies were done by a psychologist (KVdB) and a sociologist (FK), the broader research team consisted of GPs (MV and RR) and psychiatrists (GD and MD) as well. Furthermore, we need to be aware that our findings from the nominal groups may have been influenced by the summary of the focus groups we provided at the beginning of the nominal groups, and that the selection of participants and the Belgian context have a strong impact on all our findings. A final limitation that applies to these studies is the fact that no other stakeholders (e.g., patients, psychologists, social workers, nurses, …) were involved, potentially leaving us with an incomplete picture at the moment.

## Conclusions

Notwithstanding the abovementioned limitations, we strongly believe that the present study provides a basis for the development and installation of a collaborative care paradigm in Belgian mental healthcare. Our in-depth analyses resulted in a clearer, more detailed view about what may impede or facilitate the collaboration amongst professionals, which may give rise to concrete adaptations (on different levels) in current practice. In short, technological advancements, deontological clarity, financial reimbursement of psychotherapy provided by psychologists, and adapted nomenclature (reimbursement medical acts) are considered necessary conditions for collaborative care, but none of them is met for the moment. Yet, we believe that these numerous and interesting proposals could only be put into practice when practitioners’ attitude of mind changes as well. At present, we think they may be insufficiently aware of the necessity of working together and its added value for patients and health care providers. This may include an important task for education, managers and policy makers.

## Footnotes

We first used convenience sampling, but a number of the invited participants was not willing to participate. In order to obtain sufficiently large samples, we then asked the invited participants whether they knew other physicians that were interested in the topic of study.

We corrected the scores for group size in order to make them mutually comparable; the scores of the Dutch speaking participants to the nominal groups were multiplied by 1.5, because the number of French speaking participants (n = 9) was 1.5 times larger than the number of Dutch speaking participants (n = 6). We then calculated the percentage of all points each proposition received (priority; * 100/(18 participants * 15 points each)), and the percentage of the total number of votes (popularity; * 100/(18 participants * 5 votes each)) for each proposition.
